# Implementation of short incubation MALDI-TOF MS identification from positive blood cultures in routine diagnostics and effects on empiric antimicrobial therapy

**DOI:** 10.1186/s13756-017-0173-4

**Published:** 2017-01-14

**Authors:** Robin Köck, Jörg Wüllenweber, Dagmar Horn, Christian Lanckohr, Karsten Becker, Evgeny A. Idelevich

**Affiliations:** 1 Institute of Medical Microbiology, University Hospital Münster, Domagkstr. 10, 48149 Münster, Germany; 2 Present address: Institute of Hospital Hygiene, University of Oldenburg, European Medical School Oldenburg-Groningen, Rahel-Straus-Str. 10, 26133 Oldenburg, Germany; 3 Pharmacy Department, University Hospital Münster, Albert-Schweitzer-Campus 1, 48149 Münster, Germany; 4 Department for Anaesthesiology, Intensive Care Medicine and Pain Therapy, University Hospital Münster, Albert-Schweitzer-Campus 1, 48149 Münster, Germany

**Keywords:** Antibiotic stewardship, MALDI-TOF MS, Sepsis, Diagnostics, Blood culture

## Abstract

**Background:**

Results of blood culture (BC) diagnostics should be swiftly available to guide treatment of critically ill patients. Conventional BC diagnostics usually performs species identification of microorganisms from mature solid medium colonies. Species identification might be speed up by using matrix-assisted laser desorption ionization time-of-flight (MALDI-TOF) mass spectrometry (MS) of biomass from shortly incubated solid media.

**Methods:**

This single-center analysis compared the applicability of MALDI-TOF-based species identification from shortly incubated cultures in laboratory routine vs. conventional diagnostics and assessed its effects of on empiric antibiotic therapy.

**Results:**

Median time between detection of BCs as “positive” by incubators and further processing (e.g. microscopy) was 6 h 21 min. Median time between microscopy and result reporting to the ward was 15 min. Including 193 BCs, MALDI-TOF from shortly incubated biomass resulted in significantly faster (*p* > 0.001) species identification. Species results became available for clinicians after a median of 188 min (231 min for Gram-positive bacteria, 151 min for Gram-negative bacteria) compared to 909 min (*n* = 192 BCs) when conventional diagnostics was used. For 152/179 bacteremia episodes (85%) empiric antibiotic therapy had already been started when the microscopy result was reported to the ward; microscopy led to changes of therapies in 14/179 (8%). In contrast, reporting the bacterial species (without antibiogram) resulted in therapeutic adjustments in 36/179 (20%). Evaluating these changes revealed improved therapies in 26/36 cases (72%).

**Conclusions:**

Species identification by MALDI-TOF MS from shortly incubated subcultures resulted in adjustments of empiric antibiotic therapies and might improve the clinical outcome of septic patients.

**Electronic supplementary material:**

The online version of this article (doi:10.1186/s13756-017-0173-4) contains supplementary material, which is available to authorized users.

## Background

Taking blood cultures (BC) is one of the most important components of diagnostics performed for critically ill patients. As the mortality of septic patients is highly dependent on an accurate therapeutic approach during the early phase of the infection, treatment follows the general principle “*frapper fort et frapper vite*” (as postulated by Paul Ehrlich in 1913) [1]. This means that empiric therapy is initiated immediately using adequate doses of antibiotics covering the expected spectrum of pathogens [2]. Hence, the results of BC diagnostics will usually not be used to start treatment, but rather to re-assess whether initial empiric therapy was accurate and to adjust it, if necessary. This makes BC diagnostics a key issue of antibiotic stewardship programs aiming to de-escalate empiric broad-spectrum antibiotics and improve the rational use of antimicrobials [3].

However, a disadvantage associated with culture-based BC diagnostics is that it follows the “biological” clock rate of microbial growth but not the clock speed determined by the clinical progress of a severe infection. This hampers early adjustment of empiric antimicrobial therapies to diagnostic findings and leads to, first, increased morbidity or mortality of severely ill patients, and second, an extended empiric use of broad-spectrum antibiotics [4].

As a result, many recent studies have assessed the technical effectiveness of culture-independent BC diagnostic methods with the aim of identifying DNA of the causative species in the blood sample more rapidly [5–10]. However, PCR approaches are still expensive and have drawbacks regarding sensitivity and specificity [11, 12]. An alternative is to conventionally incubate BC bottles in an automated system and use matrix-assisted laser desorption ionization time-of-flight mass-spectrometry (MALDI-TOF MS) directly from BC bottles in which microbial growth was indicated by the automated system [13]. This approach is also valuable, but requires more laborious and costly processing of the BC in the microbiological laboratory.

Therefore, Idelevich et al. recently evaluated a simple alternative method for species identification: it was demonstrated that MALDI-TOF performed from “immature” biomass growing on solid media shortly (2–4 h) after inoculation with broth from a positive BC bottle, successfully identified the growing microorganisms [14]. This can be done in all laboratories where MALDI-TOF MS is available and does not increase consumable costs compared with conventional BC diagnostics (as species diagnostics has to be done anyway) [15].

While Idelevich et al. assessed the “technical” accuracy of this method, its feasibility in clinical, microbiological lab-routine has not been described. Moreover, it is unclear to what extent clinicians use the faster species information provided by MALDI-TOF MS from shortly incubated cultures for adjustments of empiric antibiotic therapy, because species information is initially provided without antibiograms. Therefore, we assessed these two issues in this retrospective study. The results shall allow for conclusions whether implementing MALDI-TOF based species identification from shortly incubated biomass has effects on empiric antimicrobial therapy.

## Methods

This analysis retrospectively assessed the effects of a change in microbiological BC diagnostics. The study was performed at the Institute of Medical Microbiology, University Hospital Münster (UHM), Germany. The laboratory offers microbiological service routinely from 7:30 a.m. to 6:30 p.m. (Monday through Friday), 7:30 a.m. through 1:00 p.m. (Saturday) and 9:00 a.m. through noon (Sunday). The institute provides service almost exclusively for the UHM, which is a maximum care university hospital with about 1400 beds in Northwestern Germany. The facilities of the institute are located on the UHM campus, i.e. the distance of the microbiological laboratory to all clinics, wards and other facilities of the hospital does not exceed one kilometer.

### Conventional BC diagnostics

Conventional BC diagnostics comprised the following routine procedures:

- BC bottles (BD BACTEC™ PLUS media) were transported from the wards to the microbiological laboratory. After arrival in the laboratory they were placed in an automated BC incubation system (BD BACTEC™ 9240). If growth in a BC bottle was indicated by the automated system, the bottle was immediately processed and streaked onto solid media (Columbia and chocolate blood agar, Schaedler agar additionally for anaerobic culture bottles). Solid media were immediately placed in an incubator and a Gram-stain was performed. The result of the Gram stain was immediately communicated to a physician on the respective ward (by phone call) and was reported to the ward as a preliminary finding electronically.
- Further routine diagnostic procedures were as follows: species identification was done via MALDI-TOF MS and antibiotic susceptibility testing (mostly) via VITEK 2 automated system. Susceptibility testing was initiated from “mature” colonies growing on solid media in the afternoon (for BC bottles detected before 9 h in the morning) or the next day (for all BC bottles detected after 9 h); species identification was done from mature colonies the next day for all BCs.

### Diagnostic changes

MALDI-TOF MS based species identification from immature biomass growing on solid media was implemented as follows: Step a.) described for conventional diagnostics remained unchanged. In step b.) the solid media inoculated with material from the positive BC bottle were visually evaluated for the first time approximately 2-3 h after start of incubation. As soon as growth of biomass (rather mature “colonies”) was visible on the agar plates, species identification was performed from these “young” cultures using MALDI-TOF MS. As soon as the species identification result was available (criteria for reliability as mentioned in [14]), it was reported to the ward. If no growth was visible, or no successful identification was achieved, the next visual evaluation was performed after further 2-3 h of incubation, followed by MALDI-TOF MS in case of visible growth. Further identification attempts after longer incubation were only performed in individual cases at the discretion of technologist or clinical microbiologist. In addition, standardized susceptibility testing (using VITEK-2) was initiated.

### Data assessment

We assessed all positive BCs from 01.01.2013–30.06.2013 (before diagnostic change) and 01.01.2014–30.06.2014 (after diagnostic change). We considered all BC bottles (filled with blood, other body fluids were excluded). BCs fulfilling the following criteria were then removed from the dataset: i.) mixed cultures (i.e. >1 pathogen in one bottle, removed due to uncomplete or unreliable MALDI-TOF results), ii.) detection of fungi and anaerobic bacteria (removed due to slow growth), iii.) detection of coagulase-negative staphylococci, corynebacteria and propionibacteria (as we aimed to assess and evaluate the effects of MALDI-TOF on empiric therapy, the BCs were removed due to unclear clinical relevance and unclear effects on empiric therapy), iv.) consecutive cultures of the same patient (i.e. if the same pathogen was detected in more than one BC of the same patient, only the culture which was first indicated as “positive” by the BC incubator remained in the dataset).

For all BCs remaining in the final dataset, the following parameters were assessed from the laboratory software (OpusL, OSM, Essen, Germany) and the electronic patient record (Orbis, AGFA Healthcare, Bonn, Germany), where they are routinely recorded:

1. Date and time of a documented Gram stain result (i.e. the time is recorded when the result is entered in the computer system),
2. Date and time of the first report of a microscopic result to the ward (i.e. the time of the phone call is actively recorded by the person communicating the result),
3. Date and time of successful species identification (i.e. the time is recorded when the species name is entered in the lab software),
4. Date and time of the species available on the ward (i.e. the time when the finding appears in the electronic patient record, which is after entering it in the lab software (see point 3 and after electronic validation).
5. For all cultures after implementation of MALDI-TOF MS from shortly incubated cultures, we additionally assessed antibiotic therapy of the patient for a time period starting two days before the BC became positive and ending after reporting the species result to the ward. The quality of therapeutic adjustments attributable to the intervention was evaluated within a local antibiotic stewardship team, including an intensive care clinician, microbiologists and a pharmacist. An adjustment was considered rational when the new therapy was more likely to target or more efficient to treat the detected microorganism than the previous therapy.

Statistical differences were assessed using Chi-Square or *t*-test (Epi Info™, version 7.2, CDC Atlanta, USA); *p* < 0.05 was considered significant.

## Results

### Infrastructural parameters

In the half-year period before the diagnostic change 1185 BC sets with microbial growth from 544 patients were retrieved compared with 1132 BC sets from 540 patients after implementation of MALDI-TOF MS from shortly incubated cultures. After clearing the dataset (with respect to criteria i. to iv. see Methods), we analyzed data for 192 BCs before and 193 BCs after the diagnostic change, respectively (*p* = 0.62). Of all 385 BCs, 117 (30%) were indicated as “positive” by the BC incubator during routine service times of the microbiological laboratory and 268 (70%) outside service hours. Overall, the median time between bacterial growth reported by the BC incubation system and microscopy was 381 min (mean 410 min, range 6 min-1188 min). There was a major difference depending on whether the BC bottle was flagged “positive” during the service time of the laboratory or not; during service time: median 48 min (mean 65 min, range 6 min-1123 min) vs. outside service time: median 547 min (mean 561 min, range 18 min-1188 min), *p* < 0.001). The median time (mean, range) between microscopy and report of the result to the ward was 15 min (44 min, 0–1386 min).

### Species identification time using conventional diagnostics vs. MALDI-TOF MS from shortly incubated cultures in clinical microbiological lab-routine

After implementation of the new concept for processing BCs in the laboratory, the time needed to report a species identification result (after initial microscopy) was significantly reduced (Table 1), even though this study partly included BCs for which MALDI-TOF MS was not carried out the same day. This effect was more prominent for Gram-negative (median 2.5 h) than for Gram-positive microorganisms (median about 4 h, Table 1).

**Table 1:** Time until species identification before and after implementation of MALDI-TOF MS from shortly incubated cultures

Bacterial pathogen	Before^a^ (in minutes; median (mean; range))	After^a^ (in minutes; median (mean; range))	*P*
All bacteria	909 (776; 2–1439)	188 (342; 0–1439)	<0.001
Gram-negative	811 (942; 9–1438)	151 (277; 36–1433)	<0.001
Gram-positive	849 (742; 2–1439)	231 (400; 0–1439)	<0.001

^a^time between microscopy and availability of species identification result before and after implementation of MALDI-TOF MS from shortly incubated cultures in laboratory routine

### Effects of species report on antimicrobial therapy

**Fig. 1:**
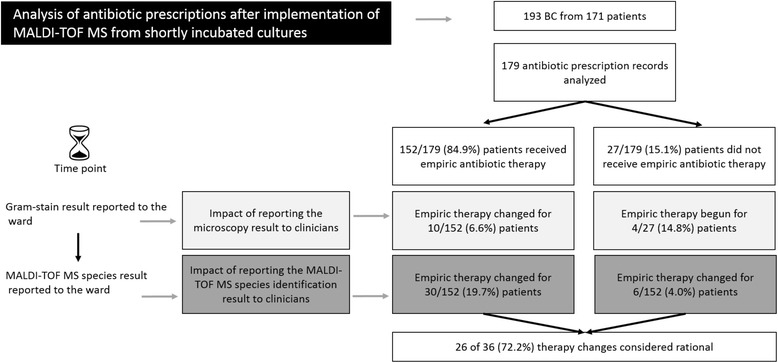
Effects of MALDI-TOF MS species identification on empiric antimicrobial therapy

**Fig. 2:**
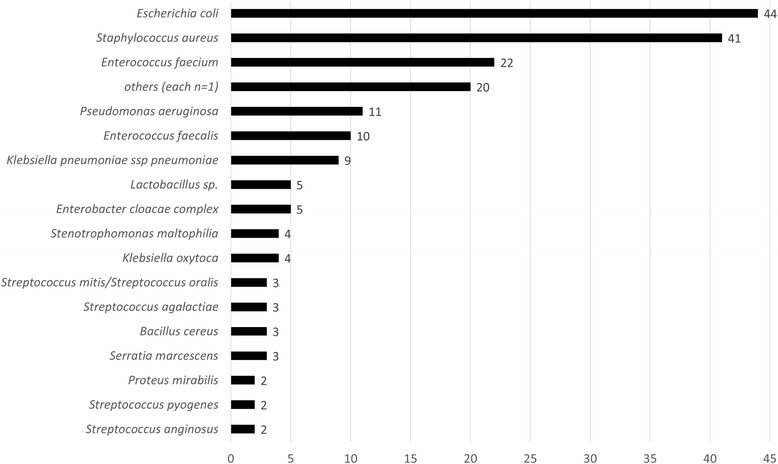
Pathogens in blood cultures included (*n* = 193). Microorganisms summarized under “others (each *n* = 1)”: *Haemophilus influenza*e, *Lactobacillus gasseri, Citrobacter freundii, Acinetobacter baumannii, Brevibacterium casei, Bacillus licheniformis, Salmonella* serovar Typhi*, Proteus vulgaris group, Enterococcus casseliflavus, Bacillus simplex, Streptococcus gallolyticus, Raoultella ornithinolytica, Streptococcus dysgalactiae subsp. equisimilis, Rothia dentocariosa, Rothia mucilaginosa, Streptococcus pneumoniae, Listeria monocytogenes, Streptococcus sanguinis, Moraxella osloensis, Pantoea agglomerans*

For the time after implementation of MALDI-TOF MS from shortly incubated cultures we analyzed antibiotic therapies for the patients with bacteremia (Fig. 1). The species distribution for microorganisms detected in the 193 BCs included in the intervention phase is shown in Fig. 2. The 193 BCs were derived from patients on nephrology wards (*n* = 35, including patients in an emergency department), hemato-oncology wards (*n* = 35), cardiology wards (*n* = 20), gastroenterology wards (*n* = 19), surgical intensive care units (ICU) (*n* = 26), pediatric wards (*n* = 18), general-, heart- and trauma-surgical wards (*n* = 15), neurology wards (*n* = 9), transplantation wards (*n* = 7), urology wards (*n* = 4), orthopedic wards (*n* = 3), wards for radiotherapy (*n* = 2), dermatology and ENT wards (each *n* = 1). The 193 BCs were taken from 172 different patients. If patients were included more than once, this was not due to consecutive cultures (see methods), but due to more than one bacteremia episode of this patient in which different bacteria were identified. For 179 of the BCs (from 158 patients), we were able to analyze the antibiotic prescriptions initiated for the respective patients (Fig. 1). The remaining patients were discharged or had died when the BC was detected positive. For 152 of the 179 bacteremia episodes (85%) an empiric antibiotic therapy was already initiated at the time of the initial contact with the ward (i.e. report of microscopy result).

Overall, after reporting the microscopy results antibiotic therapies were changed in 14/179 (8%) patients (Fig. 1). For none of these 14 cases the antimicrobial therapy was later adjusted again after the species identification result became available (before results of susceptibility tests were ready).

Overall, rapid reporting of the species identification result (without antibiogram) caused adjustments in empiric therapies of 36/179 (20%) bacteremia episodes. The mean time between reporting of species results to the ward and documented change of antibiotic therapy was 168 min (median 186 min, range 0–434 min).

Evaluating these 36 bacteremia episodes showed that the adjustments led to a better therapy in 26/36 cases (72%). Criterion for evaluating an adjustment as reasonable was that the identified species was more likely targeted with the antibiotic used than the previously used therapy (evaluations for single cases are shown in the supplementary material, Additional file 1: Table S1). Among the 36 cases in which adjustments of antibiotic treatment were performed after species identification, therapeutic changes were more often performed, if *S. aureus* was reported, as compared to all other pathogens (13/41 vs. 23/152, *p* = 0.03).

## Discussion

The major aim of this study was to compare species identification times for microorganisms from BCs using a conventional diagnostic approach versus MALDI-TOF MS based species identification from shortly incubated biomass. Technically, the applicability, validity and microbiological accuracy of this method has been demonstrated before in a pilot study [14]. In this study, we found that the results of the pilot study held true in daily routine. Performing MALDI-TOF MS of immature cultures resulted in a significant reduction of the median time until species identification for bacteria from BCs. Particularly for fast growing Gram-negative microorganisms, the median time until species identification was reduced to less than three hours. However, compared with our pilot studies the mean time until availability of species results was slightly higher (5.9 h for Gram-positive cocci and 2 h for Gram-negative rods in the study by Idelevich et al. vs. 6.7 h for Gram-positive bacteria and 4.6 h for Gram-negative bacteria in this study) [14]. This is because in contrast to the pilot study, this data assessment includes all BC cultures even if MALDI-TOF MS was not performed on the same day (e.g. for cultures detected in the late afternoon or on weekends where the service hours of the laboratory were restricted). These results are important as they show that the pilot studies do not markedly overestimate the benefits of MALDI-TOF MS of shortly incubated cultures when this technique is applied in microbiological routine diagnostics.

The application of PCR-based BC diagnostic tests has also resulted in a significant reduction of the time until species identification [16] and, partly, results were available even faster. However, major advantages of the diagnostic approach evaluated here are that it can be done without any additional consumable costs and that it leads to cultivated microorganisms ready for further characterization, in particular susceptibility testing. The efforts regarding re-organizing workflows of technical personnel and additional hands-on time was limited in our hands and the intervention was easy to implement in diagnostic routine. However, it should be noted that this study was done in a microbiological laboratory serving a single university hospital. This limited the daily number of BC bottles, which had to be processed and facilitated communication of the results to the wards. In regional labs with larger catchment areas and service for several facilities, the implementation of this diagnostic approach might be more challenging. However, recent and future developments in the field of laboratory automation including “smart” incubators and automated growth detection may facilitate the approach reported here.

Besides the positive effects of performing MALDI-TOF MS from immature biomass on the speed of diagnostics, we also found that our lab infrastructure limits the achievements made [5, 17, 18]. As the majority of BC bottles were reported “positive” by the automated incubator outside the service hours of the laboratory, the median time until further processing of a BC bottle (i.e. microscopy and plating on solid media) was >6 h. Besides offering longer service hours, another option to solve this problem might be the installation of BC incubators in areas of the hospital available for clinical personnel so that BC bottles can be continuously loaded into the systems [19]. The second infrastructural parameter assessed in this study was the time between performing microscopy and report of this result to ward. We are convinced that the 15 min interval observed is a reasonable. Outliers (>1000 min for reporting the microscopy results) were due to cases in which initial microscopy failed to identify bacteria in the Gram stain where they were actually present [20].

Besides evaluating the applicability of MALDI-TOF MS-based diagnostics in laboratory routine, we aimed to assess whether knowledge of the species (even without an antibiogram) led to adjustments of antibiotic therapies. We found that the clinicians changed empiric antibiotic therapies in 20% of all cases after communication of the species test result. This was more frequent than adjustments made based on microscopy results alone (8%). Hence, MALDI-TOF MS had an important clinical effect. On the other hand, a majority of antibiotic therapies remained unchanged. This could be explained by, first, adherence to well-designed guidelines for empiric therapies covering the reported species, second, difficulties of the treating physicians to correctly interpret the species report (without an antibiogram) with respect to how to adjust empiric therapies in a way that they are more accurate and sophisticated, or third, disregarding the diagnostic finding. Detailed evaluations of the quality of single therapeutic adjustments were difficult, as other diagnostic findings than BC results, such as allergies to antibiotics, data for organ insufficiencies and the overall prognosis of the clinical case must be considered. This is a major limitation of the retrospective study design. However, assessing the quality of adjustments after species identification in a local antibiotic stewardship team revealed that the majority of adjustments was appropriate. Clinicians particularly made adjustments for *S. aureus* bacteremia (32%), even if at this stage, antibiograms were not yet available. This might be due to the fact that at the UHM all patients are screened at admission for nasopharyngeal carriage of MRSA and, hence, MRSA bacteremia, which is mostly caused by strains colonizing the nares prior to infection [21], is rather unlikely, if a negative screening result from the nares is available. Moreover, the proportion of MRSA on all *S. aureus* isolates from BCs at our institutions followed the nationally declining trend in Germany and was <15% in 2014 (EARS-net, http://ecdc.europa.eu/, own data not shown). Interestingly, we found that in 11/13 *S. aureus* bacteremia cases (and 72% of all cases in which adjustments of antibiotic therapies were made) the modified therapy included the microorganism better the initial empiric regimen. Delport et al. recently reported positive clinical effects of performing MALDI-TOF MS from shortly incubated colonies. They observed in a pediatric patient collective that antibiotics were earlier optimized and the patients even had a favorable outcome and a shorter length of stay [22].

## Conclusions

Overall, more rapid species diagnostics led to adjustments of empiric therapies in 20% and these adjustments improved calculated therapies in 72%. Therefore, the local antibiotic stewardship program shall focus on improved communication of the more rapid species results, maybe starting with a special emphasis of *S. aureus* bacteremia cases.

## Additional file

Additional file 1: Table S1. Evaluation of adjustments of antibiotic therapies made by clinicians directly after species identification and before availability of an antibiogram. (DOC 55 kb)
