# Supplementary material for: Implementation of short incubation MALDI-TOF MS identification from positive blood cultures in routine diagnostics and effects on empiric antimicrobial therapy
Source: Antimicrob Resist Infect Control. 2017 Jan 14;6:12. doi: 10.1186/s13756-017-0173-4 (PMC5237541; doi:10.1186/s13756-017-0173-4)
Supplement: Additional file 1: Table S1. — Evaluation of adjustments of antibiotic therapies made by clinicians directly after species identification and before availability of an antibiogram. (DOC 55 kb) [file 13756_2017_173_MOESM1_ESM.doc]

**Additional file 1**

**Table S1. Evaluation of adjustments of antibiotic therapies made by clinicians directly after species identification and before availability of an antibiogram**

| **Pathogen detected** | **Adjustment and evaluation^a^** | **Adjustment considered reasonable^?a^** |
| --- | --- | --- |
| *Enterococcus faecalis* | Started AMP (no antibiotics before). | Yes |
| *Enterococcus faecium* | VA added to MER. | Yes |
| *Enterococcus faecium* | LNZ added to MER and colistin. | Yes |
| *Escherichia coli* | Started PIP/TAZ (no antibiotics before). | Yes |
| *Escherichia coli* | Started PIP/TAZ and CIP (no antibiotics before). Stopped CIP the following day (after antibiogram). | Yes |
| *Escherichia coli* | Started CTX (no antibiotics before). Escalated to MER the following day (after antibiogram: isolate was ESBL-producer). | Yes |
| *Escherichia coli* | Replaced CIP by PIP/TAZ; calculated effectiveness higher. | Yes |
| *Escherichia coli* | Changed AMO/CL to PIP/TAZ; calculated effectiveness higher. | Yes |
| *Klebsiella pneumoniae* | CTX instead of CFX and CLI; calculated effectiveness higher. | Yes |
| *Listeria monocytogenes* | Started AMP (no antibiotics before). | Yes |
| *Pseudomonas aeruginosa* | MER replaced by CTZ as known MER-resistant *P. aeruginosa* in rectal swab; however, BC isolate was CTZ-resistant. | Yes |
| *Staphylococcus aureus* | Started VA and GEN (no antibiotics before); no MRSA-screening result available. | Yes |
| *Staphylococcus aureus* | Added CFX to MER (which was continued); negative MRSA-screening result available; CFX more effective than MER. | Yes |
| *Staphylococcus aureus* | Started CFX and FOS (no antibiotics before); negative MRSA-Screening result available; known MSSA at catheter exit site. | Yes |
| *Staphylococcus aureus* | MER and LNZ instead of CTZ; over-escalation as negative MRSA-screening result available, but CTZ ineffective against *S. aureus* | Yes |
| *Staphylococcus aureus* | OXA instead of VA; negative MRSA-screening result available. | Yes |
| *Staphylococcus aureus** | LNZ added to CTZ and PIP/TAZ. Isolate was MRSA; screening results not available. | Yes |
| *Staphylococcus aureus* | Added VA to CTZ and CLI (resistant for this isolate); negative MRSA-screening available. | Yes |
| *Staphylococcus aureus* | Changed PIP/TAZ to OXA; negative MRSA-screenings available, known MSSA in wound swab. | Yes |
| *Staphylococcus aureus* | Changed PIP/TAZ to OXA; negative MRSA-screenings available. | Yes |
| *Staphylococcus aureus* | Changed PIP/TAZ and VA to OXA and RIF; negative MRSA-screenings available. | Yes |
| *Staphylococcus aureus* | Changed PIP/TAZ and VA to OXA, negative MRSA-screenings available. | Yes |
| *Stenotrophomonas maltophilia* | Started CIP (no antibiotics before). | Yes |
| *Stenotrophomonas maltophilia* | Added MOX to VA. | Yes |
| *Streptococcus dysgalactiae* | Changed CIP to AMO/CL. | Yes |
| *Streptococcus pyogenes* | Added PEN G to MER and daptomycin (the latter two were stopped the next day). | Yes |
| *Enterococcus faecalis* | Added VA to PIP/TAZ; over-escalation. | No |
| *Enterococcus faecalis* | Added VA to PIP/TAZ; over-escalation. | No |
| *Escherichia coli* | Changed PIP/TAZ to MER/VA. Negative ESBL-screenings available. | No |
| *Haemophilus influenzae* | Changed CTX to MER; no difference expected for calculated therapy. | No |
| *Klebsiella pneumoniae* | Changed PIP/TAZ to CTX, no other diagnostics available; no difference expected for calculated therapy. | No |
| *Lactobacillus sp.* | MER und TEICO changed to TGC; stopped again after two doses; previous calculated therapy more effective | No |
| *Pseudomonas aeruginosa* | Stopped LNZ and continued CTZ, GEN. LNZ could have been stopped after microscopy already; not attributable to intervention. | No |
| *Rothia mucilaginosa* | Switched to MER and VA; before: PIP/TAZ and GEN; over-escalation | No |
| *Staphylococcus aureus* | VA and RIF added to PIP/TAZ and GEN; negative MRSA-screening results available; over-escalation | No |
| *Staphylococcus aureus* | VA added to PIP/TAZ; negative MRSA-Screening results available; over-escalation | No |

^a^ reasonability of the adjusted therapy as consented after evaluation in a local antibiotic stewardship team. Methicillin-resistant *S. aureus* (MRSA); all other *S. aureus* were methicillin-susceptible (MSSA). AMO/CL, amoxicillin/clavulanate; AMP, ampicillin; CFX, cefuroxime; CIP, ciprofloxacin; CLI, clindamycin; CTX, ceftriaxone; CTZ, ceftazidime; ESBL, extended-spectrum β-lactamase; GEN, gentamicin; LNZ, linezolid; MER, meropenem; MOX, moxifloxacin; OXA, flucloxacillin; PEN G, penicillin G; PIP/TAZ, piperacillin/tazobactam; RIF, rifampicin; TEICO, teicoplanin; TGC, tigecycline; VA, vancomycin.
